# Long term survival after coronary endarterectomy in patients undergoing combined coronary and valvular surgery – a fifteen year experience

**DOI:** 10.1186/1749-8090-3-15

**Published:** 2008-03-26

**Authors:** Sanjay Kumar, Sandeep Agarwala, Charlie Talbot, R Unnikrishnan Nair

**Affiliations:** 1 Department of Cardiothoracic surgery, Yorkshire heart Centre, Leeds General Infirmary, Leeds, UK

## Abstract

**Background:**

Coronary Endarterectomy (CE) in patients undergoing coronary artery bypass graft (CABG) surgery has been shown to be beneficial in those with diffuse coronary artery disease. There are no published data on its role and benefit in patients undergoing more complex operations. We present our experience with CE in patients undergoing valve surgery with concomitant CABG.

**Materials and methods:**

Between 1989 and 2003, 237 patients underwent CABG with valve surgery under a single surgeon at our institution. Of these, 41 patients needed CE. Data was retrospectively obtained from hospital records and database. Further follow-up was obtained by telephone interview. All variables were analyzed by univariate analysis for significant factors relating to hospital mortality. Morbidity and long term survival was also studied. There were 29 males and 12 females with a mean age of 67.4 ± 8.1 and body mass index of 26.3 ± 3.3. Their mean euroscore was 7.6 ± 3.2 and the log euro score was 12.2 ± 16.1.

**Results:**

Thirty-two patients were discharged from the intensive therapy unit within 48 hours after surgery. Average hospital stay was 12.7 ± 10.43 days. Thirty day mortality was 9.8%. Six late deaths occurred during the 14 year follow up. Ten year survival was 57.2% (95% CL 37.8%–86.6%). Three of the survivors had Class II symptoms, with one requiring nitrates. None required further percutaneous or surgical intervention. We compared the result with the available mortality figure from the SCTS database.

**Conclusion:**

Compared to the SCTS database for these patients, we have observed that CE does not increase the mortality in combined procedures. By accomplishing revascularization in areas deemed ungraftable, we have shown an added survival benefit in this group of patients.

## Introduction

Coronary endarterectomy (CE), first described by Bailey et al in 1957, has been shown to benefit patients with advanced coronary atheroma by providing complete revascularization. [1] The safety and long-term efficacy of the procedure, although controversial, has been demonstrated in earlier studies. [2,3]

There have been no studies on the effect of endarterectomy in patients undergoing valve surgery with concomitant coronary bypass. This study was aimed at auditing the mortality and morbidity of patients undergoing combined valvular and coronary artery surgery with additional CE. We also looked at the symptomatic relief and long-term survival in this group of patients.

## Materials and methods

Patients who underwent coronary artery bypass combined with a valve procedure from 1988 to 2003 at a University teaching hospital by a single consultant were identified from the database. Patient data was gathered retrospectively from the case notes and operation records. Of the total 237 patients, 41(29 male and 12 female) had CE in addition to the coronary bypass and a valve procedure. Each of these patients had either a single, double or triple valve repair/replacement with a bioprosthesis or a mechanical prosthesis and had single or multiple coronary grafts with either vein and/or left internal mammary artery.

The decision to perform endarterectomy was based on the preoperative angiograms and intraoperative findings. They included arteries with 100% proximal occlusion with no distal lumen or subtotal occlusion with diffuse atheroma. In some patients more than one coronary artery were endarterectomised.

CPB with cooling to 30 degrees Celsius was established using standard aortic and right atrial cannulations. Myocardial protection was effected by intermittent cold blood cardioplegia antegradely through aortic root and proximal end of completed vein graft, and in some cases retrogradely. The topical myocardial cooling was used in all cases. The valve repair/replacements were done using the standard techniques. CE was done by opening the diseased coronary artery directly over the plaque and then by developing a plane between the adventitia and the plaque by hydro-dissection with a handheld syringe with cold saline and 20 French Abbocath^R ^(Abbott Laboratories). Adequate distal clearance was confirmed by a tapered thinned out distal segment of intima at the end of the atheromatous plaque. In selected cases a further distal arteriotomy was performed for completion of endarterectomy in cases of broken atheroma and in these cases the distal incision was closed with a vein patch.

All patients were anticoagulated for 6 months with Warfarin until 2002, when we started using a combination of antiplatelet agents for the aftercare of endarterectomy. These patients currently receive 300 mgs of Aspirin per rectally 6 hours after the procedure and are started on regular Aspirin 75 mgs and Clopidogrel 75 mgs daily from the first postoperative day. Clopidogrel is continued for 6 months postoperatively and Aspirin lifelong. Patients in atrial fibrillation or those with mechanical valves are still anticoagulated with Warfarin and aspirin.

Demographic variables, preoperative symptom status, co-morbid risk factors, operative details and postoperative events indicating morbidity and mortality were recorded. All patients were followed up at regular intervals in the outpatient clinic and the latest follow up was obtained by telephone interview at the start of the study with regards to their current symptom status, need for nitrates, hospital readmissions, and re-interventions.

All the data were stored on the database and were analyzed using SPSS 11.0. Continuous variables were expressed as mean ± SD and categorical variables were expressed as absolute numbers. All variables were analyzed with univariate analysis to determine whether any single factor influenced hospital mortality. Wilcoxon rank-sum test (for variables recorded on a continuous scale) or Fisher's exact test (for categorical variables) were used. Variables that achieved a P value less than .05 were considered significant. Attempt was made to analyze significant variables by multivariate analysis using logistic regression to evaluate independent risk factors for hospital mortality after surgical intervention. However in view of small number of study patients the data was too sparse to allow any such model to be constructed.

Survival data were analyzed by using the standard Kaplan Meier actuarial technique for estimation of survival probabilities.

## Results

Data on 41 patients, who had CE while undergoing coronary bypass with valve surgery, was analyzed for factors associated with in-hospital mortality and postoperative survival. The postoperative morbidity and symptom relief at follow up were also noted.

### Factors associated with in-hospital mortality

Four patients died within the first 30 days after surgery. Variables, which might have been associated with mortality, were identified and the association tested for statistical significance, using either the Wilcoxon rank-sum test (for variables recorded on a continuous scale) or Fisher's exact test (for categorical variables).

Factors, which were statistically significant at the 5% level, were EuroSCORE (p-value 0.016), logEuro (p-value 0.019), severity of angina (0.012), and the presence of a pacemaker (p-value 0.007). The p-values for all the variables considered are listed in table 1 and 2.

**Table 1:** Demographic Characteristics

**Variable**	**Patients**	**Number dead**	**P value**
Total number of patients	41	10(4***)	0.302
Males	29	7(4***)	
Females	12	3(0***)	
Age	67.4 ± 8.1**		0.086
BMI	26.3 ± 3.3**		0.583
Euroscore	7.6 ± 3.2**		0.016*
Log Euro	12.2 ± 16.1**		0.019*
Severity			
Stable angina	34	1	0.012*
Unstable angina	7	3	

*Significant at P < 0.05

** Mean ± 1 Standard Deviation

*** In hospital mortality

**Table 2:** Variables studied for in hospital mortality

**Risk Factor**	**N**	**Dead**	**P Value**
Congestive Cardiac Failure			
Yes	25	3	1.000
No	16	1	
Past Myocardial Infarct			
Yes	19	2	1.000
No	22	2	
Redo Surgery			
Yes	6	1	0.483
No	35	3	
Cardiomegaly on X-ray			
Yes	19	3	0.321
No	22	1	
Previous Cardiological Intervention			
Yes	4	1	0.348
No	37	3	
Neurological Dysfunction			
Yes	5	1	0.418
No	36	3	
Diabetes			
Yes	8	0	0.569
No	33	4	
Hypercholesterolaemia			
Yes	19	2	1.000
No	22	2	
Hypertension			
Yes	29	2	0.567
No	12	2	
Hypothyroidism			
Yes	1	0	1.000
No	40	4	
Smoking			
Current smoker	09	1	0.645/1
Non smoker	32	3	
Family history of IHD			
Yes	16	2	0.637
No	25	2	
Gastrointestinal			
Yes	4	1	0.348
No	37	3	
Renal			
creatinine<160 mg/dl	40	3	0.098
creatinine >300 mg/dl	1	1	
Respiratory			
Yes	3	0	1.000
PVD			
Yes	8	0	0.569
No	33	4	
Cerebrovascular			
Yes	7	1	0.542
No	34	3	
Rhythm			
Sinus rhythm	34	3	0.542
Others	7	1	
Pacemaker			
Yes	2	2	0.007*
No	39	2	
Nitrates			
Yes	1	0	1.000
No	40	4	
IABP			
Yes	1	1	0.098
No	40	3	
Priority			
Elective	38	3	0.271
Urgent	3	1	

*Significant at P < 0.05

A higher EuroSCORE was associated with increased mortality. The patients who survived had a median EuroSCORE of 7, while those who died had a median EuroSCORE of 10, giving an observed difference of 3. The 95% confidence interval for this difference was 1–11.

A higher LogEuro score was also associated with increased mortality. The median LogEuro for the two groups were 6.95 (survivors) and 16.55 (deaths), giving an observed difference in medians of 9.6 with a 95% confidence interval of 1.59–72.25.

Of 4 patients with unstable angina 3 died, while only 1 of the 37 patients with stable angina died. The observed odds ratio (i.e. the odds of dying for patients with unstable angina divided by the odds of dying for patients with stable angina) was 24.75, while the 95% confidence interval for this odds ratio was 1.38–1321.

Presence of a permanent pacemaker had an extremely low p-value. Only 2 patients had pacemakers, and both these patients died.

Of the four patients who died in hospital only two had peri-operative infarct. One died of cerebro-vascular accident and the other due to sepsis and multi-organ failure. The in-hospital mortality rate observed in this study was 9.8%.

Several cardiac and intraoperative variables were also recorded and table 3 and 4 show the p-values measuring the association between the operating characteristics and mortality. Several of these p-values are small.

**Table 3:** Cardiac Variables

**Variable**	**N**	**Dead**	**P value**
Extent of Coronary Disease			
Single Vessel	4	0	0.184
Double Vessel	13	2	
Triple Vessel	24	2	
Left Main Stem			
Yes	2	1	0.188
No	39	3	
PA Wedge			
<20 cm	29	1	0.068
>20 cm	12	3	
Valve requiring surgery			
Aortic	20	2	1.000
Mitral	18	2	
Double (aortic + mitral)	03	0	
LV Function			
Poor	10	2	0.446
Moderate	13	1	
Good	18	1	
Number of distal anastomoses			
Single	4	0	0.233
Double	15	3	
Triple	16	0	
More than triple	6	1	
Vessel requiring endarterectomy			
RCA	31	2	0.245
LAD	6	1	
Multiple	4	1	
Valve procedure			
Repair	14	1	1.000
Replacement	27	3	

**Table 4:** Operative Variables

**Variable**	**N**	**Dead**	**P Value**
Cross Clamp Time	94.5+24.1		0.947
Re exploration			
No	36	0	1.000
Yes	5	4	
Peri operative Infarct			
Yes	3	2	0.021
No	38	2	
Low Cardiac output			
Yes	9	4	0.001*
No	32	0	
Ionotropes			
Yes	27	4	0.280
No	14	0	
Vasoconstrictors			
Yes	10	3	0.039*
No	31	1	
IABP			
Yes	7	4	0.0003*
No	34	0	
Arrhythmia			
SR	21	1	0.203
AF/SVT	18	2	
VF/VT	2	1	
Arrest			
Yes	2	2	0.007*
No	39	2	
Temporary Pacing			
Yes	16	1	1.000
No	25	3	
Ventilation duration			
<48 hr	33	2	0.165
>48 hrs	8	2	
PPM			
Yes	3	1	0.271
No	38	3	

* Significant at P < 0.05

Post operative infarct (p-value 0.021), low cardiac output (p-value 0.001), cardiac arrest (p-value 0.007), use of vasoconstrictors (p-value 0.039) and use of IABP (p-value 0.0003) were associated with an increased risk of mortality. Morbidity following these operations was also recorded (table 5).

**Table 5:** Morbidity

**Variable**	**N**
Neurological Complication	
Yes	4
No	37
Renal impairment requiring filter	
Yes	1
No	40
Gastrointestinal Complications	
Yes	1
No	40
Sternal Wound Infection	
Yes	2
No	39
Tracheostomy	
Yes	5
No	36
Sepsis	
Yes	1
No	40
Multi-organ failure	
Yes	1
No	40
ITU Stay	
<48 hours	32
>48 hours	9
Hospital Stay	12.7 + 10.43
Readmission to ITU	1
Re intervention	
Medical	1
Surgical	0

**Figure 1:**
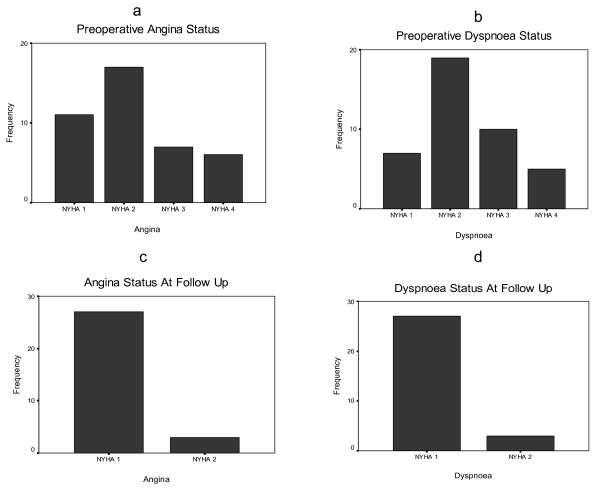
(a-d): Bar Charts comparing the symptom status before the surgery and at follow up.

Preoperative symptom status in terms of angina and dyspnoea was also compared. The bar charts figure 1(a–d) illustrate the preoperative symptom status and the status at follow up.

### Long-term survival

**Figure 2:**
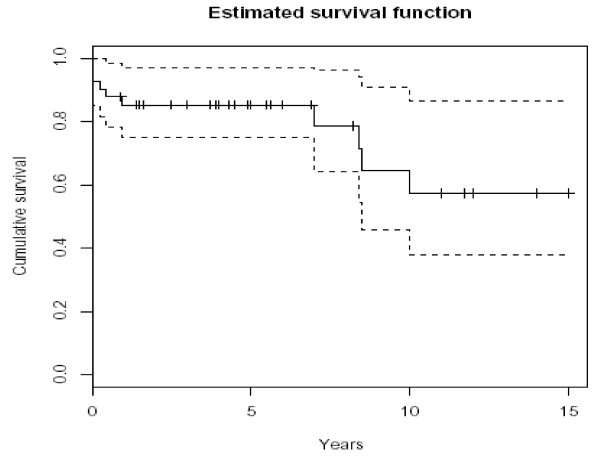
Kaplan-Meier Survival curve showing the estimated survival probabilities.

The long-term survival of the 41 patients was studied. During the recorded follow-up time (between 0 and 15 years), a total of 10 deaths occurred including those immediately following the operation. A Kaplan-Meier survival curve showing the estimated survival probabilities and associated 95% confidence intervals is given in figure 2. The "latest" death occurred 10 years after the operation. The estimated survival rate at 10 years (or longer as no further deaths occurred) is 57.2% with a 95% confidence interval of 37.8%–86.6%.

All the late (post-hospitalization) deaths have not been confirmed to be due to causes related to the coronary bypass surgery. If any of these deaths were in fact due to other causes, the actual estimated survival could have been higher. One died due to endocarditis and another due to congestive cardiac failure. Cause of death in 4 of late deaths could not be confirmed.

## Discussion

CE as a means for achieving complete revascularization has been practiced since 1950's. Bailey et al first performed it in 1956 at Hahnemann Hospital in Philadelphia. [1] Since then, various techniques have been described including the open and closed method. Carbon dioxide gas, laser, cardioplegia infusion, and other techniques have been used to separate the plane of atheroma to facilitate endarterectomy. [4-6]

The initial enthusiasm has been dampened by the reported increase in mortality and morbidity following these procedures and poorer long-term graft patency in endarterectomised vessels. [7,8] One possible caution in CE is that the removal of distal atheroma has to be complete as otherwise partially removed plaques could result in regional ischemia and perioperative infarction. There is no uniform agreement amongst surgeons in the field of CE. Those with surgical expertise in this operation seem to do more whereas others tend to graft distally or in certain cases do not attempt to graft severely atheromatous arteries. Sequential grafting is appropriate when the recipient coronary artery have a reasonable lumen. In extenstive atheromatous this may not give satisfying results.

However, the changing patient population over last decade has forced surgeons to look up at the surgical option of CE again. Over the last few years, many reports suggest that the initial impressions about the mortality and morbidity may not have been true and CE may be a good way of providing better symptomatic relief and survival in this group of patients with more diffuse disease.

Recent literature has looked at the issues of mortality and morbidity in patients undergoing CE in isolated coronary bypass operations. Typically reported rates have been between 0%–15% over the last fifteen years with postoperative myocardial infarction rates being 5–25% [9]. Asimakopoulos et al have reported mortality of 3.6% in their study group of patients, which was not statistically different from the mortality in the control group, suggesting that with improved safety in modern surgery mortality is not increased in patients requiring CE. Survival rates of 71–92% at five years have been shown in previous studies, however long term survival in these groups of patients is not known. [10]

With increasing use of off pump coronary bypass, isolated groups have reported CE in this subgroup with good initial results. Eryilmaz et al have reported no mortality with 100% patency rates on angiographies done at one year and have further strengthened the concept of need and justification for complete revascularization in patients with severe diffuse atherosclerotic disease and poor LV function. [11]

Mortality in the group of patients undergoing combined valve surgery with coronary bypass has traditionally been higher. In this group of patients the general approach is to keep the operations as simple as possible with minimum being done to keep the operative time less and thereby hoping to keep the mortality down.

The association of valve disease (particularly of mitral valve) requiring concomitant surgery in patients undergoing bypass and endarterectomy has been reported to be an independent, risk factor for increased mortality. In this same study aortic valve replacement was associated with increased risk of peri-operative MI. [12]

This is a retrospective analytical observation of the outcome of surgery specially in patients who had CE in addition valve surgery. The data goes back to 1988 before the advent of statistical markers such as EuroScore. [13] Hence it is difficult to compare this group with conventional CABG and valve surgery.

The United Kingdom Cardiac Surgical Register for the financial year ending 2001 reported mortality rate of 7.8% (224 patients died out of a total of 2,881) for patients undergoing combined valve surgery and coronary bypass [14]. Comparing these figures with our data on patients also undergoing endarterectomy (mortality rate of 9.8%), there was no evidence of a statistically significant increase in mortality for the patients undergoing endarterectomy (p-value of 0.56 using Fisher's exact test). The observed odds ratio was 1.28 (95% CL 0.33–3.62). The median postoperative hospital stay reported in the register for valve procedures combined with coronary bypass was 8 days [14] which compares favorably with our group of patients who had a median stay of 7 days.

Comparing the results between the endarterectomised and non-endarterectomised vessels is controversial. There is a general agreement on the fact that endarterectomy is done only for vessels which are near totally occluded with poor run offs, thus implying more severe disease process. In this scenario, is it justified comparing an endarterectomy group with one, which does not undergo endarterectomy ? Perhaps, in these situations, results are best judged by the long-term symptom free status and survival rather than the patency of the grafts per se.

Livesay et al in their series of endarterectomies noted the fact that the patients in the endarterectomy group differed significantly from others in gender, diabetes low ejection fraction and increased number of bypassed vessels, thus implying sicker patients with more severe disease. In their study they did not find significant differences in the mortality, long-term survival, symptom benefit and freedom from reoperations especially in the group of patient who were operated in the later era with improved techniques for myocardial protection and better anticoagulation drugs. They believe that in patients with more diffuse disease this forms a good option to provide as good results as those with less severe disease. [2]

The issue of increased risk with left sided endarterectomies has not been conclusively proven and some studies have shown satisfactory results [15]. Silberman S et al have shown that patients requiring left anterior descending artery endarterectomy are at an increased risk of operative mortality. [16] Sundt et al have shown that there is no difference in the incidence of perioperative MI or dysarrhythmias in between various vessels endarterectomised. They had a 75–76% actuarial survival at 5 years with 74% being angina free. Only 3% had required a redo surgery in long term follow up [17]. In our group of patients we also did not note any difference in mortality in different vessels endarterectomised.

Ferraris et al have earlier shown a reduced long-term angiographic patency in their group of patients. In this group the patency was 40% for the endarterectomised vessels in the study group as compared to 58% patency for the non-endarterectomised vessels in the same group. The patency rate was reported to be 65% in the control group [7]. This discrepancy in the patency between the control group and the non-endarterectomised vessels of the study group may reflect the more severe disease in the study group in the first place. This study may also have included a bias due to the fact that it was done by selecting retrospectively, the patients who had undergone repeat catheterization. This would have possibly included only those patients with problems and symptoms and would not have taken into account the total number of endarterectomised patients during the study period that probably did not require a re-angiography as they were symptom free. Thus it would have picked up a falsely high number of blocked grafts wrongly attributed to endarterectomy. Also endarterectomy was done only for patients with unsuitable vessel for grafting; thus without endarterectomy and grafting the area would be ischemic in 100% of the patients but by doing the procedure it provided revascularization for at least 40% of the patients over a long follow up period.

Brenowitz et al stratified operative mortality and long term survival according to the presence of additional risk factors such as left ventricular dysfunction, repeat operation, diabetes, female sex, and age over 70. The results showed increased operative mortality and morbidity; however better survival and clinical status with continued graft patency justified this approach in patients with diffuse coronary artery disease many of whom would otherwise have been deemed inoperable. [3]

A 2.3%mortality and 85% patency at 15.4 months have been reported by Frazone et al[18], Doss et al, however, claim better patency with the plaque bridging anastomoses [19] but this type of anastomoses may not be suitable for patients with no distal runoff.

Djalilian and Shumway have shown patency rates of 83% at 1 year and 75% at average interval of 37 months. Their incidence of MI was 5% and mortality of 3%. [9]

Keogh et al have used angioscopy to assess the completeness of endarterectomies. [20] They have shown that this can be used for confirming the completeness of the endarterectomy without increasing ischemic time. In their group of patients they showed that the small side branches were not compromised.

Incomplete endarterectomy may have a snow-plough effect leading to the occlusion of side branches. The increased thrombogenicity of the raw area may also contribute to higher occlusion rate and post op infarction. Better results can be achieved by ensuring completeness of the endarterectomy and by better anticoagulation management. The variable results noted in different series with improved results in later ones might be reflective of this fact.

In a study by Tasdemir et al, the actuarial survival at 7 years after endarterectomy was better than simple patch reconstruction and bypass (94%v/s 74.8%, p = 0.008) reflecting a complete removal of the atherosclerotic material and complete revascularization in the endarterectomy group of patients. This study also concluded that the life expectancy, functional capacity, and working status of patients who underwent endarterectomy was comparable to those in the control group with possibly less extensive disease. [21]

Factors determining the outcome of operative correction of valvular abnormalities combined with coronary artery bypass grafting are still incompletely defined. Increasing age, female sex, renal failure, advanced New York Heart Association class, left ventricular function, mitral regurgitation, coronary disease, cardiac arrrhythmia and the operative ischemic time are all important predictors of adverse clinical outcome after combined valve operations and coronary artery bypass grafting. Five and 10 year survival probabilities of 74% (95% CL of 71% – 78%) and 43% (95% CL 36% – 50%), respectively have been reported [22]. Mitral valve disease is associated with poorer long-term outcomes with 10 year actuarial survival rates of 33% (95%CL 22% – 47%) in the ischemic group and 52% (95%CL 42%–64%) in the degenerative group.

Survival after mitral valve surgery and CABG is determined by the extent of coronary disease, ventricular dysfunction and by the success of the valve procedure. Etiology of mitral valve regurgitation has relatively little impact on late outcome.

Mullany CJ et al in their study have shown that delineation of coronary artery disease and revascularization decreases the mortality of aortic valve surgery. [23] Early improvement in ejection fraction, influenced by coexistent coronary artery disease and gender-associated factors, significantly affects subsequent survival. [24]

The extent of coronary artery disease is an important predictor for long-term survival after combined valve and coronary bypass operations. [25] Complete revascularization in this group of patients we believe is even more important for better long-term survival.

In our group we had 57.2% (95%CL 37.8% – 86.6%) survival at 10 years (which includes deaths from all causes), which is better than that reported in literature. Thus endarterectomy might be indirectly providing a survival advantage and should be seriously considered in appropriate patients with diffuse disease.

## Conclusion

The long-term survival in patients with combined valvular and coronary artery disease is determined by the extent of coronary disease. On comparison with the published outcome data from SCTS database, the results from this retrospective observational study has shown that CE does not increase the mortality or morbidity in this small complex cohort of patients. CE in diffuse coronary artery disease facilitates complete revascularization and hence possibly offers survival benefit. In view of the small numbers in our group, this requires to be validated by a multicentre study with larger number of patients.
